# Malaria Control and Elimination,^1^ Venezuela, 1800s–1970s

**DOI:** 10.3201/eid2010.130917

**Published:** 2014-10

**Authors:** Sean M. Griffing, Leopoldo Villegas, Venkatachalam Udhayakumar

**Affiliations:** Centers for Disease Control and Prevention, Atlanta, Georgia, USA (S.M. Griffing, V. Udhayakumar);; ICF International, Calverton, Maryland, USA (L. Villegas);; Centro de Investigación de Campo Dr. Francesco Vitanza, Bolívar, Venezuela (L. Villegas)

**Keywords:** malaria, Plasmodium vivax, Plasmodium falciparum, parasites, chloroquine, DDT, Arnoldo Gabaldón, epidemiology, control, eradication, elimination, Venezuela

## Abstract

Venezuela had the highest number of human malaria cases in Latin American before 1936. During 1891–1920, malaria was endemic to >600,000 km^2^ of this country; malaria death rates led to major population decreases during 1891–1920. No pathogen, including the influenza virus that caused the 1918 pandemic, caused more deaths than malaria during 1905–1945. Early reports of malaria eradication in Venezuela helped spark the world’s interest in global eradication. We describe early approaches to malaria epidemiology in Venezuela and how this country developed an efficient control program and an approach to eradication. Arnoldo Gabaldón was a key policy maker during this development process. He directed malaria control in Venezuela from the late 1930s to the end of the 1970s and contributed to malaria program planning of the World Health Organization. We discuss how his efforts helped reduce the incidence of malaria in Venezuela and how his approach diverged from World Health Organization guidelines.

## Abstract 2

Control programs used during the 20th century are case studies for current programs.

Venezuela had the most human malaria cases in Latin America before 1936. During 1891–1920, malaria was endemic to >600,000 km^2^ of Venezuela; deaths from malaria substantially reduced the population during 1891–1920 (*1*). No pathogen, including influenza virus (1918 pandemic), caused more deaths than malaria during 1905–1945. Early malaria epidemics had mortality rates of 60–70 deaths/1,000 persons; rates were as high as 531 and 1,125 deaths/100,000 persons in Carabobo and Cojedes States in 1941 (*2*).

Venezuela can be divided into 3 zones: central (Los Llanos; plains), southern (Guayana), and northern (Costa-Cordillera; coast–mountain range) (Figure 1). Los Llanos has grassy plains intersected by rivers that flood and abut jungles. This zone contains 36% of Venezuela and bodies of still water in which vectors breed. In the early 20th century, 20% of the population lived in Los Llanos and had the greatest malaria prevalence; however, no large epidemics occurred there. In northern regions, malaria was considered hyperendemic based on spleen indexes (Table), which occasionally reached 100. In southern regions, spleen indexes were <50. The malaria vector was *Anopheles darlingi* mosquitoes, one of the most efficient neotropical vectors. In Venezuela, these mosquitoes bite throughout the night or adapt to human behavior. Their larvae require clear water (*5*). These mosquitoes were absent in southwest regions near the Apure River, which were free of malaria (*3*).

**Figure 1:**
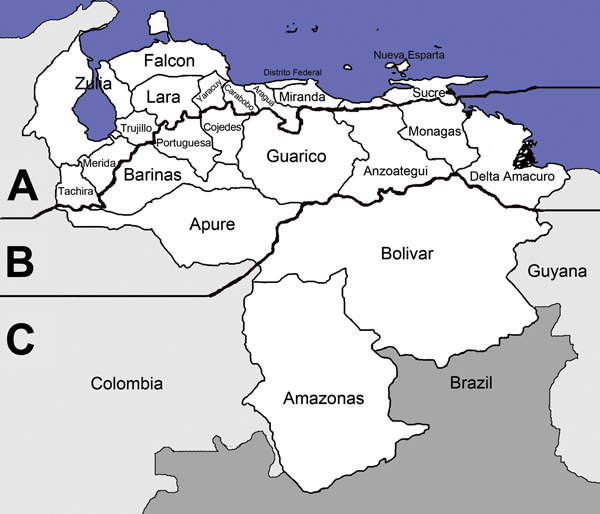
Three zones of Venezuela used by Arnoldo Gabaldón for treatment of malaria: A) Costa-Cordillera, B) Los Llanos, and C) Guayana (*3*).

**Table:** Commonly used malaria terms, Venezuela

Term	Definition
Spleen index or spleen rate	Point prevalence of persons with a palpably enlarged spleen (splenomegaly), which is strongly associated with malaria in many countries, including India and Venezuela. Although this term is not an exact measurement of malaria infection, it is considered an indicative public health measurement in tropical countries. The reference measurement in Venezuela was 5%, where malaria was not present (*1*).
Endemicity ratio	Lowest spleen index observed in a 5-year period divided by 5, which is the assumed reference value for this index. This ratio was complemented by the ratio of epidemicity, in which the numerator was replaced by the greatest spleen index over a 5-year period (*2**,**4*).
Index of infection	Prevalence of persons with malaria infection as determined by existing measurements, such as spleen indexes or blood smears.

Guayana, which borders Brazil, Colombia, and Guyana, has a tropical forest, patches of open country in northern regions, and a savannah plateau in southern regions. Although Guayana contains 46% of Venezuela, in the early 20th century, it contained only 3% of the population, which was concentrated around urban centers. Malaria cases typically occurred at altitudes of 500–1,000 m on the plateau, where *An. darlingi* mosquitoes predominated. Spleen indexes were usually <50. In northeastern regions, *An. darlingi* mosquitoes were absent and spleen indexes were ≈5. The southwestern border of Guayana and Colombia was free of malaria because the tannic Atabapo and Guainía Rivers kept riverine villages free of *An. darlingi* mosquitoes (*3*).

Costa-Cordillera, a coastal plain that abuts mountains, contains 18% of Venezuela. Before the 1940s, malaria epidemics followed a 5-year cycle associated with coastal invasions by *An. darlingi* mosquitoes. These cyclic increases in malaria continued until at least 1997 because of the El Niño Southern Oscillation (*6*). Early control efforts divided Costa-Cordillera into western, central, and eastern sectors. The eastern sector contained Nueva Esparta State, Caribbean islands, and Sucre State. The central sector contained valleys and mountains. The western sector contained valleys of Lake Maracaibo and the Andes Mountains (*3*).

In the 1940s, 70% of the population of Venezuela lived in Costa-Cordillera (*6*). During the 1940s and 1950s, the greatest malaria endemicity occurred where *An. darlingi* mosquitoes predominated, although rates were also high where *An. albitarsis* mosquitoes predominated. Regions with moderate endemicity typically had *An. albimanus* mosquitoes, which contributed to epidemics associated with heavy rainfall or rice cultivation because larvae require sunlit water (*2**,**5*). Coastal marshes precluded survival of *An. darlingi* mosquitoes but supported survival of *An. albimanus* mosquitoes that were tolerant of brackish water (*5*). At higher altitudes, but rarely above 500 m, malaria was transmitted by *An. pseudopunctipennis* mosquitoes (limit 1,000 m) (*3*).

In eastern Costa-Cordillera, *An. darlingi* and *An. albimanus* mosquitoes predominated in western Sucre, and *An. aquasalis* mosquitoes predominated in eastern Sucre (*3*). *An. aquasalis* mosquitoes are typically coastal vectors because they compete poorly with other *Anopheles* species and have limited predator defenses (*5*). These mosquitoes are exophilic, prefers to live outdoors, and are refractive to domicile insecticide spraying (*7*). In central Costa-Cordillera, *An. darlingi* mosquitoes were found near Lake Valencia, which had the greatest malaria prevalence. In the southern sector, *An. nuneztovari* and *An. pseudopunctipennis* mosquitoes were present in foothills and *An. albimanus* mosquitoes were present in a valley (*3*). *An. nuneztovari* mosquitoes were exophilic and resisted domicile DDT spraying (*8*). In the western sector, *An. albimanus* mosquitoes predominated in northern low-rainfall zones, and *An. darlingi* mosquitoes predominated in high-rainfall zones (*3*).

Before DDT use, most municipalities in central Costa-Cordillera and western Los Llanos had endemicity ratios <8 (Table) and mortality rates of 20–25 deaths/1,000 persons. Some municipalities had endemicity ratios of 10–15 and mortality rates of 30–50 deaths/1,000 persons (*2*).

## History of Malaria in Venezuela

During the Venezuelan War of Independence (1820s–1830s), a malaria epidemic affected armies in Los Llanos (*9*). In August 1879, Ortiz in Los Llanos reported 125 malaria cases and ≥2 deaths among ≈9,600 inhabitants. Witch doctors and charlatans complicated disease treatment. In 1880, a total of 127 cases were reported, but the number of cases gradually decreased until at least 1885 (*10*). Epidemics occurred in Ortiz during 1890–1891 (*9*).

In 1894, Dr. Santos Aníbal Dominici identified the malaria parasite in patients at Vargas Hospital in Caracas (*11**,**12*). The National Health Office and Institute of Hygiene and Chemistry, Bacteriology, and Parasitology Laboratories opened in 1911; a National Health Act was promulgated in 1912 (*12*). During the 1920s, quinine was freely distributed in some regions (*9*).

In 1926, the National Health Office began to study malaria around Lake Valencia with support from the Rockefeller Foundation. The office conducted a malaria survey during 1927–1928 and recommended spraying Paris green, draining lagoons, and cultivating surrounding fields (*9**,**13*). Malaria was widespread in Los Llanos, the lower Yaracuy Valley, and the Lake Maracaibo District, but not in the Caracas Valley or the coastal region near La Guaira. Epidemics occurred in sections of the Lake Valencia basin (*14*).

The Rockefeller Foundation started a 1-year study of malaria around the Maracay District of Lake Valencia, which included patient histories, and spleen, blood, and vector surveys. Interventions began almost simultaneously, which decreased malaria cases and quinine use. A permanent program was recommended, which included better drainage for wells, irrigation ditches, and sewers (*14*). In 1930, malaria cases increased in Maracay because of introduction of *An. darlingi* mosquitoes. In 1 area, all 500 inhabitants were infected with *Plasmodium falciparum* (*15*). Cooperation with the Rockefeller Foundation lapsed in 1932, possibly because of a backlash against foreign oil companies (e.g., the Rockefellers’ ownership of Standard Oil) (*9**,**12**,**16*). Despite the foundation’s absence, effectiveness of antimalarial treatments was studied in Guárico in 1935 (*17*).

In 1936, Dr. Enrique Tejera, formerly manager of the National Health Directorate’s Bacteriology and Parasitology Laboratory, became Minister for Health and Social Assistance. The ministry oversaw the Malaria Division, which had a budget of ≈$10 million (in 2014 US dollars). Tejera created a national public health system based on administrative medical, research, and control technique units, as advocated by the Rockefeller Foundation and the League of Nation’s Malaria Commission. He established agreements with the foundation and a scholarship program for persons from Venezuela to study at universities in the United States (*12**,**18**,**19*).

In 1936, the Law on the Defense against Malaria was modeled on laws in Argentina and passed. The law acknowledged the national threat of malaria and described comprehensive interventions at local to national levels (*20*). According to dissatisfied physicians, including Tejera, the law proposed insufficient scientific studies to inform officials on whether malaria should be controlled or eradicated. Tejera resigned rather than accept ratify law. Dominici took over and designated Gabaldón as Director of Malariology (*11**,**12*).

## The Gabaldón Era

Gabaldón, a physician, had assisted Tejera at the National Health Directorate’s laboratory during 1928–1930. He had then studied at the German Institute of Naval and Tropical Diseases and the Italian Experimental Station for the Antimalarial Battle before returning to Venezuela in 1932. He received a health science doctorate from John Hopkins University in 1935 through the Rockefeller Foundation and interned at Rockefeller University in New York City (*12*).

**Figure 2:**
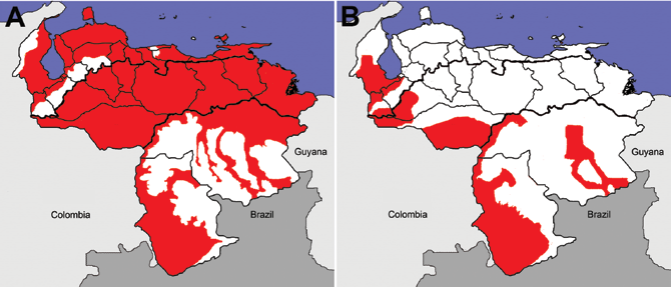
Distribution of malaria (red) in Venezuela during A) 1937 and B) 1980. This figure is qualitative because we did not have direct access to underlying data from original sources (*1*,*21*).

Under Gabaldón, the Malaria Division opened in 1936. The division had 4 sections: Epidemiology, Local Malaria Control and Quinine Distribution Commissions, Malaria Engineering, and Administration (*18*). The Malaria Division conducted an epidemiologic evaluation of malaria, vectors, and habitats and found that malaria was present throughout Venezuela (Figure 2, panel A) (*21**,**22*). It established a School of Malariology in 1937 in Maracay (*9**,**19*) and trained federal and state malaria staff, including doctors, inspectors, and engineers, during the 1940s (*9**,**19*). It also hosted the annual International Malaria and Environmental Health Course for New World malariologists (*9*).

The initial goal of the Malaria Division was to define where to apply malaria control by creating village-level maps and monitoring fumigation crews. Inspectors later managed personnel in rural areas who provided municipal diagnosis. Personnel were selected based on education and community status (*19*).

In 1937, field stations were established in towns and rural districts to monitor malaria incidence (*1*). Volunteers provided free quinine and quinacrine tablets every 7 days to febrile citizens (*1*,*2*). Blood films were examined and vectors identified at field laboratories and results were verified at central laboratories (*1*). In 1 year, 800,000 persons were treated (*2*). By 1941, the division had surveyed 8 states and planned to examine the remaining 12 states by December 1942 (*1*).

Vector control consisted of implementing sanitary engineering, including paving canals with concrete (some towns required >50 km of paving), and applying insecticides and larvicides (Paris green and pyrethrum), especially during epidemics (*2*). Larvicides were impractical without drainage to limit vector-breeding areas. Mosquito nets were widely distributed (*1*). Vector control was limited to urban areas because rural control was not economical (*1*). The main vectors were *An. albimanus* and *An. darlingi* mosquitoes, although *An. darlingi* mosquitoes were eliminated from some towns (*2*).

Gabaldón successfully experimented with pyrethrum spraying in 1940. By 1941, malaria control had been implemented in 10 cities and the index of infection (Table) was 0 in Maracay (*18*). During 1945, Gabaldón visited the United States and learned about DDT. He procured 10 kg with the support of Tejera in his capacity as governor of Carabobo. In December 1945, DDT domiciliary spraying began on a ranch in Morón, Carabobo, and eventually included 80 houses (*9*).

Indoor spraying with DDT was planned for the malarious region without preliminary trials, although initially only in northern and central Venezuela (*2*). DDT was secured through Colonel Ernest Steel, director of the Inter-American Cooperative Office of Public Health (*9*). Spraying was conducted simultaneously with antimalarial programs by using a volunteer network (*1*). Initially, 1 g of DDT/m^2^ was applied every 3 months, then every 4 months; 2 g was then applied every 6 months (*2*). Random wall scrapings were taken to verify proper spraying (*1*).

Other insecticides were also used for spraying homes. These insecticides included a benzene hexachloride/DDT mixture in areas heavily infected with triatomids. Spraying with DDT continued through a trial and error phase until the entire malarious region was covered in 1951. Challenges included weather, uneducated workers, poor supervision and transportation, and developing a team spirit (*2*). In 1946, Rockefeller University was invited to undertake malaria studies with the Malaria Division and opened a research laboratory in Maracay that focused on residual insecticide effectiveness (*23**–**25*).

Success of DDT spraying was startling. Malaria disappeared after 3–5 years without additional measures beyond occasional quinacrine use in areas where *An. darlingi* and *An. albimanus* mosquitoes predominated. The populace was stationary, which limited introduced cases and facilitated eradication. (For the remainder of the paper, the term malaria eradication, rather than the modern term malaria elimination, will be used because eradication was public health terminology for the historical period described.) Eradication in eastern regions was slower because of *An. aquasalis* and *An. nuneztovari* mosquitoes (*1*). *P. falciparum* malaria was most common, although *P. vivax* malaria predominated among children <5 years of age (*2*).

In 1946, Gabaldón proposed an Expert Committee on Malaria to the Interim Commission that would suggest future work by the World Health Organization (WHO) (*1*). Successes in Venezuela and India led the committee to conclude that “insecticides can be [used]… for a widespread attack on malaria with… a significant reduction of morbidity” (*26*). The Expert Committee defined malaria control guidelines in 1947 (*27*). Gabaldón chaired the WHO expert committee meeting and attended nearly all of its first 15 sessions (*28*).

A malaria-eradication public health network was developed by the 1950s, which WHO used as an eradication program model (*2*,*19*). By 1952, there were 590 physicians throughout central Costa-Cordillera. Physicians reported clinically diagnosed malaria cases each week to the Division of Epidemiology and Vital Statistics. The division chief also sent a letter to physicians explaining the role of the malaria eradication network and likening cases to those of yellow fever or plague (*2*).

The division emphasized microscopy confirmation of blood film findings, and medical dispensaries paid for blood films in some regions. Thick and thin blood films were examined at field laboratories and at the central laboratory, where all positive results and 10% of negative results were verified. In rural districts, after domicile pesticide spraying, health care workers obtained blood films from febrile patients or persons who had been febrile in the past week. Films were used only when physicians were absent, although the index of infection for health workers (0.3%) was nearly the same as that for case-patients (0.2%) in 1952. When malaria occurred where it been declared eradicated, an inspector and survey team measured adult vector and larval densities in areas of 5–10 km around reported case-patients. Houses were resprayed if >3 months had passed since workers’ last visit (*2*).

In the first 8 years of DDT spraying, *An. darlingi* mosquitoes and endemic and epidemic malaria were eradicated from central Costa-Cordillera, where ≈50% of the population of Venezuela lived. However, *An. aquasalis* mosquitoes were not eliminated from coastal Costa-Cordillera (0.1% of the malarious zone), where 28% of the malaria cases in Venezuela occurred (*1*). Another 50.6% of malaria cases occurred on the western border of Costa-Cordillera and Colombia (3.3% of the malarious zone), where vectors were *An. darlingi*, *An. nuneztovari*, and *An. pseudopunctipennis* mosquitoes (*1*). Control efforts were successful except near forests and banana plantations (*7*). Northern Costa-Cordillera bordering Colombia (5% of the malarious zone) had 5.6% of malaria cases, and vectors were *An. albimanus* and *An. darlingi* mosquitoes (*1*).

Eradication was not attempted in areas where distances inhibited economic control or outdoor transmission predominated. These areas included northern Costa-Cordillera along the border with Colombia, Apure and Delta Amacuro in Los Llanos, and Bolivar and Amazonas in Guyana (*2*). In Los Llanos and Guayana, 56.6% of the malarious zone contained 14.7% of malaria cases in 1952, and the main vectors were *An. albimanus*, *An. albitarsis*, and *An. darlingi* mosquitoes (*2*).

By 1954, malaria had been eliminated or was decreasing across 30% (≈180,000 km^2^) of the malarious zone (*2*). Gabaldón wrote that Venezuelan “malaria eradication…will be attained in the near future… [with]… two exceptions…. the first… two small areas… [with] out-of-doors transmission … the second… districts inhabited by nomadic and… wild Indian tribes, most… in Amazonas, Apure, Bolívar, and the Delta Amacuro” (*2*). Gabaldón refuted critics by citing successes in Argentina, Ecuador, the United States, and Venezuela (*28*). The hope was to eradicate malaria by 1955 (*29*).

However, in 1956, Gabaldón insisted that “nothing except the lack of funds should prevent the attainment of [a malaria-free Venezuela]” (*29*). Malaria reached its lowest incidence in 1959 (911 cases), and 68% of the malarious zone (407,945 km^2^) was malaria free (*1*,*21*). Gabaldón was Minister of Health during 1959–1964 and changed the Division of Malariology to the Ministry of Malariology and Environmental Health, which now included the divisions of sanitary engineering, rural water supply, rural housing, and ankylostomiasis and other helminthic diseases. He also suggested, through the Pan American Sanitary Bureau, that WHO create a registry of regions where malaria eradication was achieved (*28*).

The results of DDT spraying illustrated that primary vectors could mask contributions of secondary vectors (*28*). Spraying eliminated *An. darlingi* mosquitoes, but other vectors continued to transmit malaria (*2*). Before use of DDT, the most prevalent *Plasmodium* species was *P.* *falciparum*, followed by *P.* *vivax*, and *P.* *malariae*. Almost 40 years after introduction of DDT, *P. vivax* predominated; there was little *P. falciparum* and no *P. malariae* (*1*). In control areas, demand for quinacrine decreased. There were fewer malaria-positive blood films and death certificates that mentioned malaria or fever, and lower overall mortality rates, especially among young persons (*1*).

In 1959, febrile patients whose blood films were positive for *P. falciparum* were treated with chloroquine, followed by 4 weekly doses of chloroquine and pyrimethamine. *P. vivax* and *P. malariae* malaria was treated with chloroquine and primaquine for 3 days, then with primaquine for 11 days. Primaquine treatment was interrupted if side effects developed, and it was not given to persons >4 months of age. In the presence of DDT-refractory vectors, persons were treated with suppressive weekly or biweekly doses of pyrimethamine. Chloroquine was substituted in areas where *Plasmodium* spp. were pyrimethamine resistant (*30*).

In 1961, WHO declared malaria eradicated from 68% (407,945 km^2^) of the malaria zone in Venezuela (*1*,*31*). However, the DDT campaign ended in 1965 without eradicating malaria (*2*,*22*). Gabaldón’s successes enabled him to ignore WHO malaria strategies developed during meetings he chaired. In 1968, a WHO report found that “the concept of malaria eradication adopted by the national authorities has… and is… at variance with the [expert committee].” Against committee recommendations, Gabaldón had enlarged the eradication program to address other public health issues and no longer conducted active case detection in maintenance zones, except near zones in the attack phase. Health service staff did not view eradication as integral and were inadequately supervised. Active case detection was no longer conducted in most locations, and with passive case detection, only 30%–35% of blood films were examined, Since 1960, a total of 94 of 385 municipalities had not prepared blood films (*28*).

Venezuela declared that malaria was eradicated in some regions, although insecticide spraying continued. This declaration was in conflict with the WHO eradication definition because spraying could indicate residual endemicity. Gabaldón claimed that spraying prevented reintroduction. WHO resolved this disagreement by declaring that Venezuela was a special case of malaria eradication because it occurred before the 1960 WHO definition was developed (*28*).

Gabaldón proposed that WHO revise its global eradication strategy and include his strategies at the 1970 expert committee meeting. He concluded that permanent interruption of transmission was unachievable because of relapses and new introductions. However, if initial cases were discovered early, elimination measures could be applied without altering overall malaria eradication status (*28*). This proposal reflected his earlier shift from eliminating the reservoir of infective cases to interrupting transmission through domicile insecticide spraying and killing engorged mosquitoes (*1*). Insecticide spraying was a natural measure and applied seasonally even without adequate supervision (*28*).

Gabaldón suggested 2 levels of malaria reintroduction prevention: first-degree, which sought to prevent vector and parasite importation by proactively searching for carriers; and second-degree, which focused on limiting reestablishment of endemic malaria transmission, chiefly through pesticide spraying (*28*). He later said that first-degree prevention was ineffective and costly when applied to infected agricultural workers who moved from malarious regions to malaria-free regions (*1*). There was no need for first-degree prevention if second-degree prevention was maintained. Therefore, resources required for first-degree prevention were better spent in Latin America on permanent public health programs for transmission control, combined with preventive medicine and environmental improvement. Instead of dismantling eradication infrastructure, Gabaldón suggested that it should be converted into “vector-borne-disease control… in charge of problems that require… control measures… among environmental health activities” (*28*).

After the expert committee rejected Gabaldón’s revisions, he disassociated himself from parts of the WHO 15th report (*28**,**32*). His first-degree and second-degree prevention were mentioned. However, his assertion that second-degree prevention obviated the need to visit carrier households was not mentioned. The report emphasized integration of malaria control with health services. Gabaldón had integrated malaria eradication with preventive medicine and environmental sanitation and believed that adding medical services would be cost prohibitive. Finally, the report suggested that a region must abstain for 2 years from large-scale insecticide or mass treatment to go from the consolidation phase to the maintenance phase of eradication (*32*). Gabaldón later blamed this recommendation as the principal cause of renewed transmission in tropical countries where spraying had been correctly applied (*1*).

In 1971, the malaria-free region of Venezuela had increased to 77% (460,054 km^2^) of the malarious zone. Malaria control in malarious regions consisted of domicile spraying with DDT every 4 months (no agricultural use), as had been implemented since 1947 and would continue until 1983 (no insecticide resistance). It also included weekly mass administration of chloroquine and primaquine for <3 months in villages with monthly parasite incidences >50 per 1,000 (units were not provided) (*1*). Primaquine was probably well received because only 2% of persons sampled in Caracas in 1966 had the glucose-6-phosphate dehydrogenase deficiency associated with poor primaquine response (*33*).

**Figure 3:**
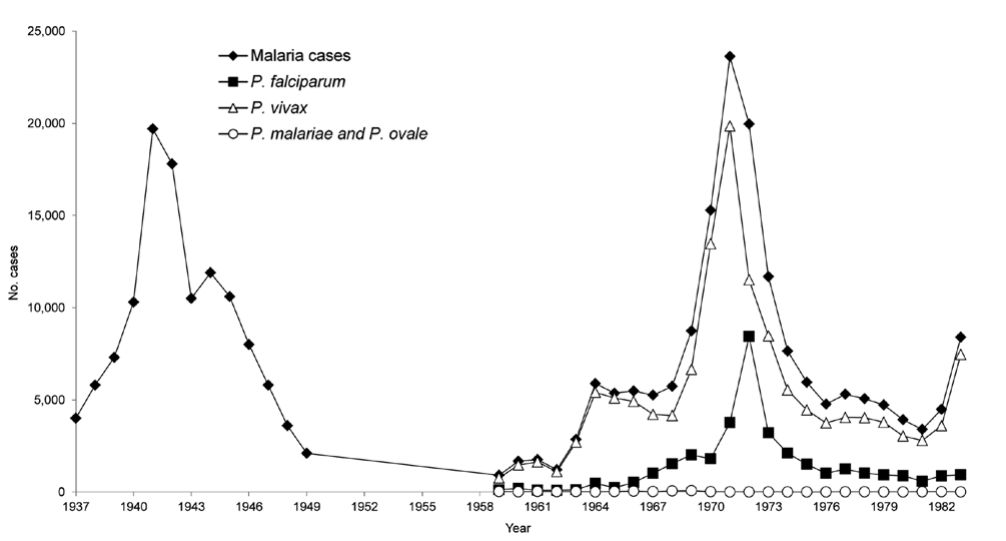
Annual malaria cases, by *Plasmodium* species, Venezuela, 1937–1983. Data for 1949 and earlier are estimates but remaining data are exact (*8**,**17**,**34*).

Malaria cases increased during the early 1970s but were decreasing when Gabaldón retired in 1973 (Figure 3) (*28*). Gabaldón noted that cases were reintroduced by agricultural laborers into malaria-free regions where insecticides were not applied. This finding led to >100 new foci per year, often near malaria-endemic areas. The foci typically involved immunologically naive populations and were easy to identify by vigilance services. Applying DDT successfully to migratory Amerindian populations and behaviorally refractory mosquitoes was difficult. Venezuela reversed the increase in malaria incidence by the late 1970s (Figure 2, panel B; Figure 3). In 1983, Gabaldón claimed that his malaria control approach empowered his eradication success (*1*). Unfortunately, malaria incidence in Venezuela increased as the 1980s began.

## Conclusions

**Figure 4:**
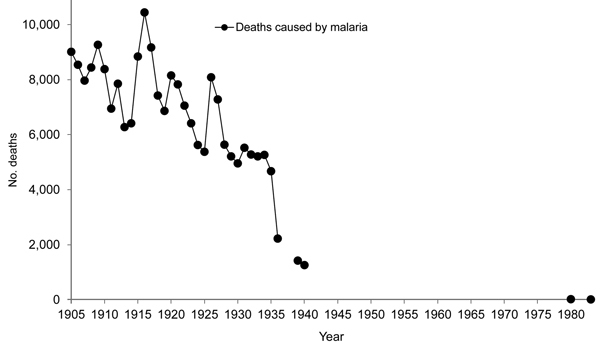
Malaria mortality rates, Venezuela, 1905–1983 (*35**,**36*). Arnoldo Gabaldón acknowledged that early malaria mortality rates in Venezuela had inherent limitations compared with rates for countries in temperate zones. The main limitations were deaths that were not registered or cases that were not diagnosed. These limitations were partially due to insufficient numbers of doctors covering the low-density populations and variations in data reporting between states and over time (*36*). Later estimates are likely of higher quality (*35*). Other mortality rates reported by Gabaldón and Berti are probably accurate because they are either generalizations or specific data that they likely considered accurate (*1**,**2*).

The early success of malaria control in Venezuela was caused by interruption of malaria transmission through systematic and integrative infection and vector control. This control included detailed knowledge of malaria epidemiology at the local level (microepidemiology); case management (diagnosis, patient treatment, and mass drug administration); mapping malaria cases; a malaria health information system updated weekly; community participation through volunteer community health workers; application of larvicides and imagocides; and sanitary engineering (housing improvement, water management). Before DDT was available, Gabaldón used these tools to reduce malaria incidence by 40% during 1941–1944 and malaria-associated deaths by 45% during 1936–1940 (Figures 3, 4) (*17*). However, DDT was a key factor in the eradication program in Venezuela when it became available in 1945.

The approach of Gabaldón to malaria eradication differs little from modern day prevention, control, and elimination, although it was implemented in a world where vector and parasite resistance were distant rumbles and governmental support was strong. However, this approach diverged from later stages of malaria eradication defined by WHO. Gabaldón integrated malaria control with sanitary engineering, rather than with clinical treatment. He also acknowledged that in a world of porous borders, malaria reintroductions would continue. Therefore, vector control would require long-term investment.
